# Laser-scanning astrocyte mapping reveals increased glutamate-responsive domain size and disrupted maturation of glutamate uptake following neonatal cortical freeze-lesion

**DOI:** 10.3389/fncel.2014.00277

**Published:** 2014-09-09

**Authors:** Moritz Armbruster, David Hampton, Yongjie Yang, Chris G. Dulla

**Affiliations:** Department of Neuroscience, Tufts University School of MedicineBoston, MA, USA

**Keywords:** freeze lesion, GLT-1, astrocyte, GLAST, glutamate

## Abstract

Astrocytic uptake of glutamate shapes extracellular neurotransmitter dynamics, receptor activation, and synaptogenesis. During development, glutamate transport becomes more robust. How neonatal brain insult affects the functional maturation of glutamate transport remains unanswered. Neonatal brain insult can lead to developmental delays, cognitive losses, and epilepsy; the disruption of glutamate transport is known to cause changes in synaptogenesis, receptor activation, and seizure. Using the neonatal freeze-lesion (FL) model, we have investigated how insult affects the maturation of astrocytic glutamate transport. As lesioning occurs on the day of birth, a time when astrocytes are still functionally immature, this model is ideal for identifying changes in astrocyte maturation following insult. Reactive astrocytosis, astrocyte proliferation, and *in vitro* hyperexcitability are known to occur in this model. To probe astrocyte glutamate transport with better spatial precision we have developed a novel technique, Laser Scanning Astrocyte Mapping (LSAM), which combines glutamate transport current (TC) recording from astrocytes with laser scanning glutamate photolysis. LSAM allows us to identify the area from which a single astrocyte can transport glutamate and to quantify spatial heterogeneity in the rate of glutamate clearance kinetics within that domain. Using LSAM, we report that cortical astrocytes have an increased glutamate-responsive area following FL and that TCs have faster decay times in distal, as compared to proximal processes. Furthermore, the developmental shift from GLAST- to GLT-1-dominated clearance is disrupted following FL. These findings introduce a novel method to probe astrocyte glutamate uptake and show that neonatal cortical FL disrupts the functional maturation of cortical astrocytes.

## Introduction

Developmental cortical malformations are a cause of focal cortical epilepsy and are often refractory to treatment (Schwartzkroin and Walsh, 2000; Crino, 2005). The freeze lesion (FL) model of cortical malformation is used to model these conditions and closely approximates human polymicrogyria (Dvorak et al., 1978; Jacobs et al., 1996, 1999). Briefly, mouse pups are anesthetized on the day of birth (P0), and a 1 by 1.5 mm copper freezing probe cooled to −50 to −60°C is applied to the exposed skull for 5 s. Following neonatal FL, a microgyrus forms and the surrounding cortical circuitry (paramicrogyral zone) generates epileptiform activity beginning at P12–14 (Jacobs et al., 1996, 1999; Andresen et al., 2014; Bell and Jacobs, 2014). In addition to changes in neuronal systems, astroglial cells are also altered in the FL model (Bordey and Sontheimer, 1998) and previous studies show that alterations in glutamate uptake occur (Campbell and Hablitz, 2008; Dulla et al., 2012). No studies, however, have investigated glutamate uptake on a single cell basis in the FL model. An important consideration in this model is that astrocytes are functionally and structurally immature at the time of FL (see review, Freeman, 2010). Therefore, developmental processes and lesion-induced changes become intermingled. How neonatal insult affects developing astrocytic glutamate uptake, both acutely after lesion and more long term, is not well understood.

Astrocytic excitatory amino acid transporters (EAATs) are the primary glutamate clearance mechanism in the CNS (Rosenberg et al., 1992). GLT-1 (EAAT2) dominates functional uptake in the mature brain while GLAST (EAAT1) plays a more prominent role in the immature brain (see review Danbolt, 2001). Loss of astrocytic glutamate uptake is pro-convulsive and neurodegenerative (Rothstein et al., 1996; Tanaka, 1997) and has been reported in diseases of cortical malformation (Boer et al., 2010). More subtle changes in astrocyte glutamate uptake may also have long term consequences on network function as astrocytes are known to regulate synaptic function (Pfrieger and Barres, 1997) and plasticity (Katagiri et al., 2001; Omrani et al., 2009). Furthermore, alterations in glutamate uptake in the neonatal cortex may affect glutamate-induced synapse formation (Kwon and Sabatini, 2011) leading to potentially permanent changes in network connectivity. Despite the importance of astrocyte glutamate uptake, surprisingly little is known about how neonatal cortical insult affects the development of this astrocytic function.

In order to examine astrocyte glutamate uptake with more specificity, we have developed a novel assay, Laser Scanning Astrocyte Mapping (LSAM). LSAM combines laser-scanning glutamate photolysis (Shepherd et al., 2003; Jin et al., 2006; Katz and Dalva, 1994) with glutamate transporter current (TC) recording. Glutamate uptake through EAATs is electrogenic and therefore TCs can be recorded in response to synaptic stimulation or photolysis of caged glutamate (Grewer et al., 2000; Bergles et al., 2002; Diamond, 2005; Grewer and Rauen, 2005). The kinetics of the TC decay are representative of the rate of clearance of glutamate (Diamond, 2005) and TCs are differentially sensitive to selective EAAT inhibitors depending on transporter sub-type composition (Arriza et al., 1994). The addition of laser-scanning glutamate photolysis to whole-cell TC recording allows focal photoactivation of MNI-glutamate to evoke TCs in a spatially and temporally precise manner. Using this tool, we examined the glutamate responsive area of individual astrocytes, TC kinetics within that glutamate-responsive area, and TC sensitivity to EAAT-specific pharmacological blockade. We report that neonatal FL increases the glutamate-responsive domain of astrocytes following FL, induces heterogeneity in intra-astrocyte TC kinetics, and alters EAAT sub-type specific pharmacological sensitivity of TCs.

## Materials and methods

### Freeze lesion surgery

Freeze lesions surgeries were performed as previously described (Dulla et al., 2012), but modified for mice. Freeze lesioned mice show similar hyperexcitability, morphological changes, and reactive astrocytosis as has been shown in the rat model (Andresen et al., 2014). Briefly, C57/Bl6 mouse pups were anesthetized via hypothermia on the day of birth (P0), a small incision in the skin over the left somatosensory cortex was made, and a 1 by 1.5 mm copper freezing probe cooled to −50 to −60°C was applied to the exposed skull for 5 s. Sham operated littermates were generated by leaving the probe at room temperature. After lesioning, the incision was resealed using surgical glue, and pups were warmed and returned to the dam. All protocols were approved by the Tufts Institutional Animal Care and Use Committee.

### Preparation of brain slices

Cortical brain slices, 400 μm thickness, containing the sensorimotor cortex were prepared from P7–10 and P26–34 C57/Bl6 mice of either sex. Mice were anesthetized with isoflurane, decapitated, and the brains were rapidly removed and placed in cold slicing solution equilibrated with 95% O_2_:5% CO_2_ (in mM): 2.5 KCl, 1.25 NaH_2_PO_4_, 10 MgSO_4_, 0.5 CaCl_2_, 11 glucose, 234 sucrose, and 26 NaHCO_3_. The brain was glued to a Vibratome VT1200S (Leica) and slices were cut in a coronal orientation. Slices were then placed into a recovery chamber containing artificial cerebrospinal fluid (aCSF, in mM: 126 NaCl, 2.5 KCl, 1.25 NaH_2_PO_4_, 1 MgSO_4_, 2 CaCl_2_, 10 glucose, and 26 NaHCO_3_ equilibrated with 95% O_2_:5% CO_2_) containing 0.5 mM sulforhodamine 101 (SR-101) for 5 min at 32°C, then equilibrated in ACSF at 32°C for 1 h. Slices were allowed to return to room temperature and used for electrophysiology.

### Field EPSP recording

Cortical brain slices were placed on an interface recording chamber maintained at 34°C superfused with oxygenated aCSF at 2 mL/min. Ascending cortical inputs were stimulated with a tungsten concentric biopolar stimulating electrode (FHC, ME) at the layer VI-white matter boundary. A recording electrode was placed in layer II-III of the cortex. Signals were recorded using glass micropipettes (resistance ≅ 1 MΩ) and acquired with DP-311 amplifier (Warner Instruments, CT) and digitized with a PowerLab 8/35 (AD Instruments, CO) using LabChart software.

### Whole-cell recording from astrocytes

Whole-cell patch clamp recordings were made from astrocytes identified in the deep cortical layers (layers IV-VI) in the paramicrogyral zone, generally within 1 mm of the freeze lesion and in isotopic areas in sham lesioned animals. Brain slices were placed into a submersion chamber (Siskiyou, OR), held in place with small gold wires, and perfused with aCSF at physiological temperature (32°C) at a flow rate of 2 mL/min. Astrocytes were identified by morphology (small, round cell bodies) and SR-101 labeling (Nimmerjahn et al., 2004) as imaged with a Cy3 filter cube (excitation 560/40 nm, emission 630/75 nm, Chroma, VT) on an Olympus BX51 microscope equipped with differential interference contrast optics. Astrocyte internal solution contained (in mM) 120 potassium gluconate, 20 HEPES, 10 EGTA, 2 MgATP, and 0.2 NaGTP. 4–12 MΩ borosilicate pipettes were utilized to establish whole cell patch-clamp recordings using a Multiclamp 700B patch clamp amplifier (Molecular Devices, CA), sampled at 10KHz using pClamp software. Once a whole-cell recording was established, cells were confirmed as astrocytes based on their passive membrane properties, low membrane resistance, and hyperpolarized resting membrane potential. Membrane properties were established by running a membrane test protocol in voltage clamp mode while holding the astrocyte at −80 mV with a 50 ms, +1 mV membrane step, averaged over 100 sweeps. This data was used to calculate R_membrane_ and R_access_. A current/voltage (I/V) curve was also generated for each cell in current clamp mode using a current injection protocol (−300 to 650 pA for 250 ms in 50 pA increments). Recorded membrane resistances were 3.7 ± 0.8, 3.98 ± 1.1, 3.1 ± 0.5, 2.8 ± 0.6 MΩ for sham neonatal, FL neonatal, sham mature, and FL mature astrocytes, *N* = 12, 8, 33, 19 cells respectively. Access resistance was 25.3 ± 4.8, 18.5 ± 3.0, 14.0 ± 1.4, 14.9 ± 1.7 MΩ for sham neonatal, FL neonatal, sham mature, and FL mature astrocytes, *N* = 12, 8, 33, 19 cells respectively. Resting membrane potential was −72.3 ± 0.9, −82.2 ± 1.6, −73.0 ± 1.3, −73.0 ± 1.6 mV for sham neonatal, FL neonatal, sham mature, and FL mature astrocytes, *N* = 12, 8, 33, 19 cells respectively.

### Astrocytic glutamate transporter recordings

**Figure 1:**
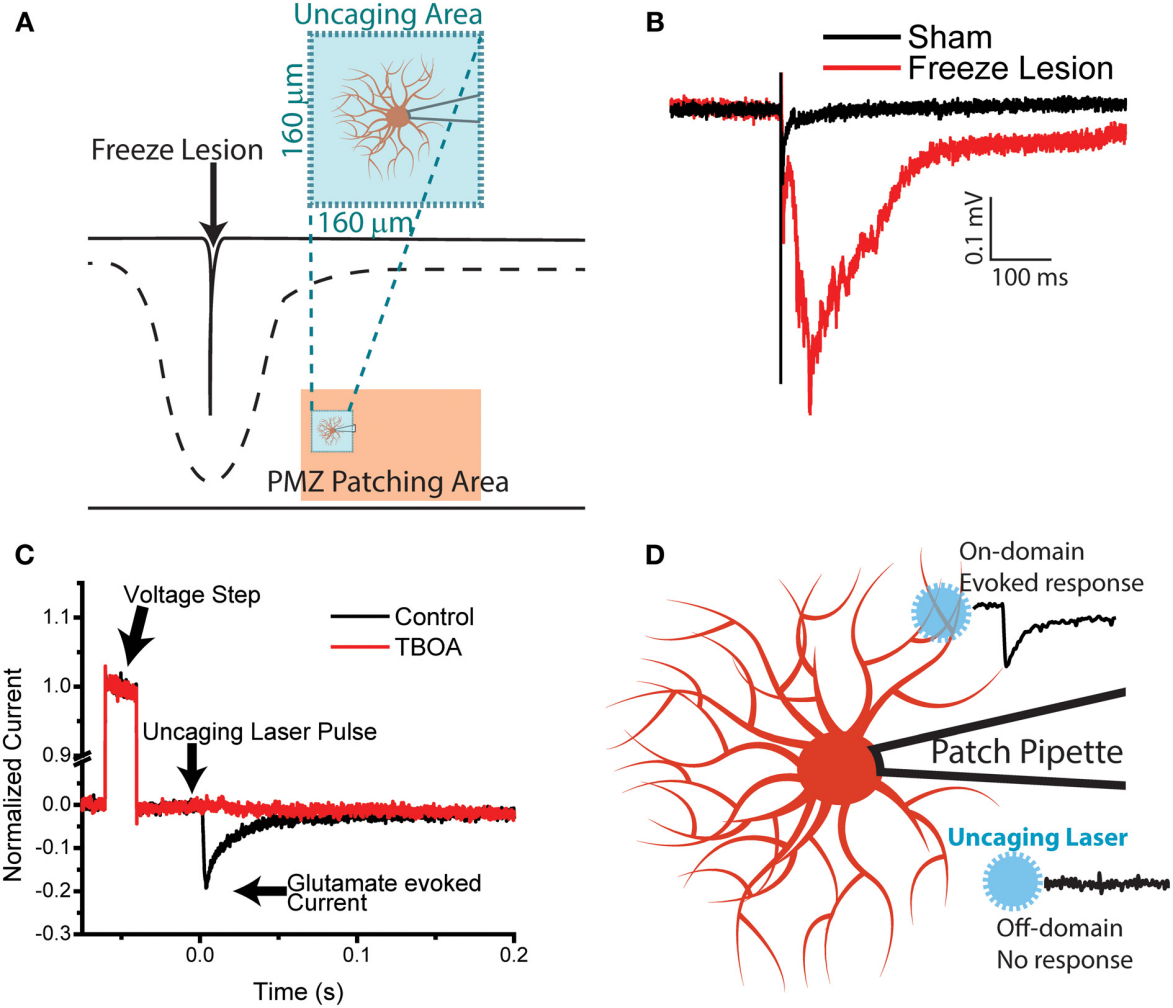
**Recording glutamate transporter currents from astrocytes following freeze lesion in a spatially defined manner. (A)** Cartoon showing an in-folding of the cortical layer structure following freeze lesion. Whole-cell recordings are made from astrocytes in the paramicrogyral zone from deep cortical layers. A 160 μm square area surrounding the patch-clamped astrocyte is then subjected to laser scanning photostimulation with MNI-glutamate present in the perfusate. **(B)** fEPSP example trace, with a stimulating electrode in the white matter of the cortex and recording in layer II–III, shows epileptiform activity following Freeze Lesion. **(C)** An example glutamate laser uncaging trace demonstrating voltage step and glutamate evoked transporter current (black). Addition of the glutamate transport inhibitor TBOA (100 μM) eliminates the glutamate transporter current (red). **(D)** In order to map the astrocytic glutamate domain of individual astrocytes, we establish a whole-cell patch configuration of a SR-101 labeled astrocyte. Using a laser-scanning photostimulation system, we spatially modulated the location of uncaging. A large glutamate evoked current is recorded when uncaging occurs within the domain of an astrocyte, while an off-domain uncaging evokes a minimal current.

In astrocytes identified by SR-101 and membrane properties, TC were recorded similar to previous studies (Diamond, 2005). Slices were perfused with aCSF containing 2 mM 4-Methoxy-7-nitroindolinyl-caged-L-glutamate (MNI glutamate, photo-activated glutamate), 10 μM SR-95531(gabazine, antagonist of GABA_A_ receptors), 20 μM 6,7-dinitroquinoxaline-2,3-dione (DNQX, antagonist of AMPA receptors), and 10 μM 3-(2-Carboxypiperazin-4-yl)propyl-1-phosphonic acid (CPP, antagonist of NMDA receptors), which was oxygenated and circulated at 2 ml/min at 34°C. TC were activated by photostimulation using a 100 mW 355 nm ultra-violet (UV) steerable uncaging laser with 10 μm full width at half maximum (FWHM) spot size (Prairie Technologies, Photoactivation System, WI). Laser power was modulated with a neutral density filter wheel and laser exposure times were controlled using a fast shutter (Sutter Instruments, CA). In voltage-clamp mode, whole-cell patch clamped astrocytes were maintained at −80 mV. To record TCs, each sweep contained a +5 mV, 20 ms voltage step, followed 40 ms later by a 1 ms UV uncaging pulse with a 50% neutral density filter (Figure 1B). Laser light was delivered via an Olympus 60×/N.A. 0.9 water immersion objective (LUMPLANFLN 60XW).

### Laser-scanning photostimulation of astrocytes

Glutamate sensitive domain mapping utilized a 10 × 10 grid of laser uncaging sites with average spacing of 16.4 ± 0.2 μm (*n* = 153 maps). The mapping protocol utilized between 200 and 300 sweeps, representing 2–3 repetitions for each uncaging location. The order of uncaging locations was randomized independently for each cell. A 650 ms delay between sweeps was utilized for laser-scanning photostimulation mapping. Following completion of a control map, 100 μM meclofenamic acid (MFA, antagonist of gap junctions) (Xu et al., 2010) or 300 μM dihydrokainate (DHK, antagonist of GLT-1 glutamate transporters) was washed on for 3–5 min before a subsequent map was recorded.

### EAAT2 reporter mice imaging

Sham and FL injured mice expressing the human EAAT2 promoter driven tdTomato (Yang et al., 2011; Morel et al., 2014) reporter were transcardially perfused with 4% paraformaldehyde. Fixed brains were sectioned at 40 μm using a Thermo Fisher Microm HM 525 cryostat. Brain sections were mounted using Vectashield (Vector Labs, CA) and imaged on a Nikon A1R confocal. The number of tdTomato positive cell bodies in of layers IV–VI and layers II-III in the PMZ were counted to measure the astrocyte density.

### Analysis

All repetitions of identical uncaging locations were averaged prior to analysis. All analysis was done offline utilizing Matlab (Mathworks, MA) or Origin (Origin Lab, MA). The TCs were calculated by modeling the membrane properties as a current divider between the membrane resistance and the access resistance with the TC being the current source. Using this model, glutamate transporter current recordings were scaled by the series resistance based on the +5 mV voltage step. Lastly in the model, recordings were scaled by the membrane conductance of the cell. These scalings correct for changes in access resistance during the recording, and for differences in membrane resistance between cells, which enables the comparison of peak and integrated TC between cells and conditions for somatic uncaging locations.

Classification of responses as On-domain or Off-domain was based on the onset time of the TC. The onset time was measured by fitting a piecewise function (f(x) = c for x<Onset_time f(x) = (x-Onset_time)^*^b+c for x≥ Onset_time) to the 5 ms surrounding the uncaging pulse using a non-linear least square fit with the Levenberg-Marquardt algorithm. On-domain onset times were characterized as less than 2.25 ms and greater than 0.75 ms from the center of the uncaging pulse with a 95% fit confidence interval of <1 ms. Based on the quality of fit criteria, this imposes a signal to noise filter in addition to the temporal filter. Based on many somatic uncaging events we determined that there was a minimum delay of 0.75 ms before the onset of the current, presumably based on the photolysis, and transport time. On-domain responses were additionally characterized as being located at the soma or on the distal processes based on the uncaging location, <20 μm between the center of the soma and the center of the uncaging location was characterized as somatic, and >20 μm was characterized as a distal uncaging location. For decay time, rise-time, peak, and integrated current measurements, all soma and all distal traces were averaged prior to analysis. T_1/2_ decay of the glutamate evoked current is the time between the peak of the evoked current and when it has decayed by half, corrected for the offset, and was calculated utilizing a 5 point moving average smoothing filter. The offset is primarily not mediated by glutamate transporters, and is present when blocking glutamate transport with TBOA (Figure 1B) (Diamond, 2005). Similarly the T_1/2_ rise time of the TC was measured between the uncaging time and the peak of the current. Integrated current was calculated based on summing the glutamate evoked current and subtracting the offset present after the glutamate current has decayed across all integrated time points.

The uncaging responsive area was calculated based upon the area spacing of the 10 × 10 uncaging grid, and the on/off cell characterization for each uncaging location. For all treatments (MFA/Laser Power/DHK), first a control map was created, followed by wash in of a drug and a second map, enabling paired comparison. The on/off-domain categorization before drug application was used for comparing traces before and after the treatment to ensure that identical locations were compared. The treatment on/off cell characterization was used to quantify changes in the glutamate responsive area. Centroids of TC were used to characterize the DHK insensitive currents. They were calculated from the uncaging until 5× the average T_1/2_ decay time constant from the peak. Two outlier cells were removed from the mature sham integrated current figure (**Figure 5C**) and one cell from the 2X power area figure (**Figure 3D**) based on the Tukey's Outlier Filter.

### Statistics

All data is presented as Mean ± s.e.m. and as noted paired or two sample *t*-tests with or without log-correction, and Analysis of Variance (ANOVA) were used as appropriate.

#### Drugs and reagents

All salts and glucose for buffers were obtained from Sigma-Aldrich unless otherwise noted. TBOA, DHK, CPP, DNQX, Gabazine (SR-95531) were obtained from Tocris, maintained as 1000× stock (TBOA, CPP, DNQX, Gabazine) or 250× stock (DHK) in DMSO (DNQX, TBOA) or in water (CPP, DHK, Gabazine). DMSO final concentration was 0.1 or 0.2% with TBOA.

## Results

### Laser-scanning astrocyte mapping

In the FL model, acute cortical brain slices generate epileptiform activity after P12–14 (Jacobs et al., 1996, 1999; Bell and Jacobs, 2014). This phenotype is mainatined in the mouse FL model (Figure 1B) (Andresen et al., 2014). Previous studies have implicated changes in astrocytes and glutamate reuptake in driving the network alterations (Bordey and Sontheimer, 1998; Campbell and Hablitz, 2008; Dulla et al., 2012). In order to characterize astrocytic changes in the FL model, we have combined whole-cell glutamate transporter currents (TCs) recording (Bernardinelli et al., 2005; Diamond, 2005), with laser-scanning MNI-glutamate photolysis. This technique, which we have termed LSAM, allows us to evoke TCs with improved spatial and temporal specificity as compared to full-field flash uncaging. Using LSAM, we set out to determine the territory from which individual astrocytes remove glutamate from the extracellular space (glutamate responsive area) and the heterogeneity of TC decay kinetics (a proxy for functional glutamate clearance) within that area (Figure 1A).

In an acute slice preparation, astrocytes were patched in a whole-cell configuration and cell identity was confirmed by SR-101 loading (Nimmerjahn et al., 2004), membrane properties, and resting membrane potential. TC were evoked by a focal (10 μm full width at half max) 1 ms 355 nm laser uncaging pulse which photolyses the biologically-inactive glutamate compound, MNI-glutamate and releases free glutamate molecules. Astrocyte glutamate transporters carry a net inward charge per molecule of glutamate translocated across the membrane (Grewer et al., 2000; Bergles et al., 2002; Grewer and Rauen, 2005); therefore, upon photolysis of MNI-glutamate, an inward current is evoked. This current was carried specifically by glutamate transporters as demonstrated by its pharmacological sensitivity to DL-threo-β-benzyloxyaspartic acid (TBOA), a broad-spectrum excitatory amino acid transporter inhibitor (Shimamoto et al., 1998), (Figure 1C). The evoked currents were similar in kinetics and pharmacological sensitivity to flash lamp evoked photolysis of MNI-glutamate (Diamond, 2005). Importantly, unlike synaptic stimulation of TCs, photolysis does not elicit neuronal action potential firing and subsequent increases in extracellular potassium levels (Callaway and Katz, 1993).

**Figure 2:**
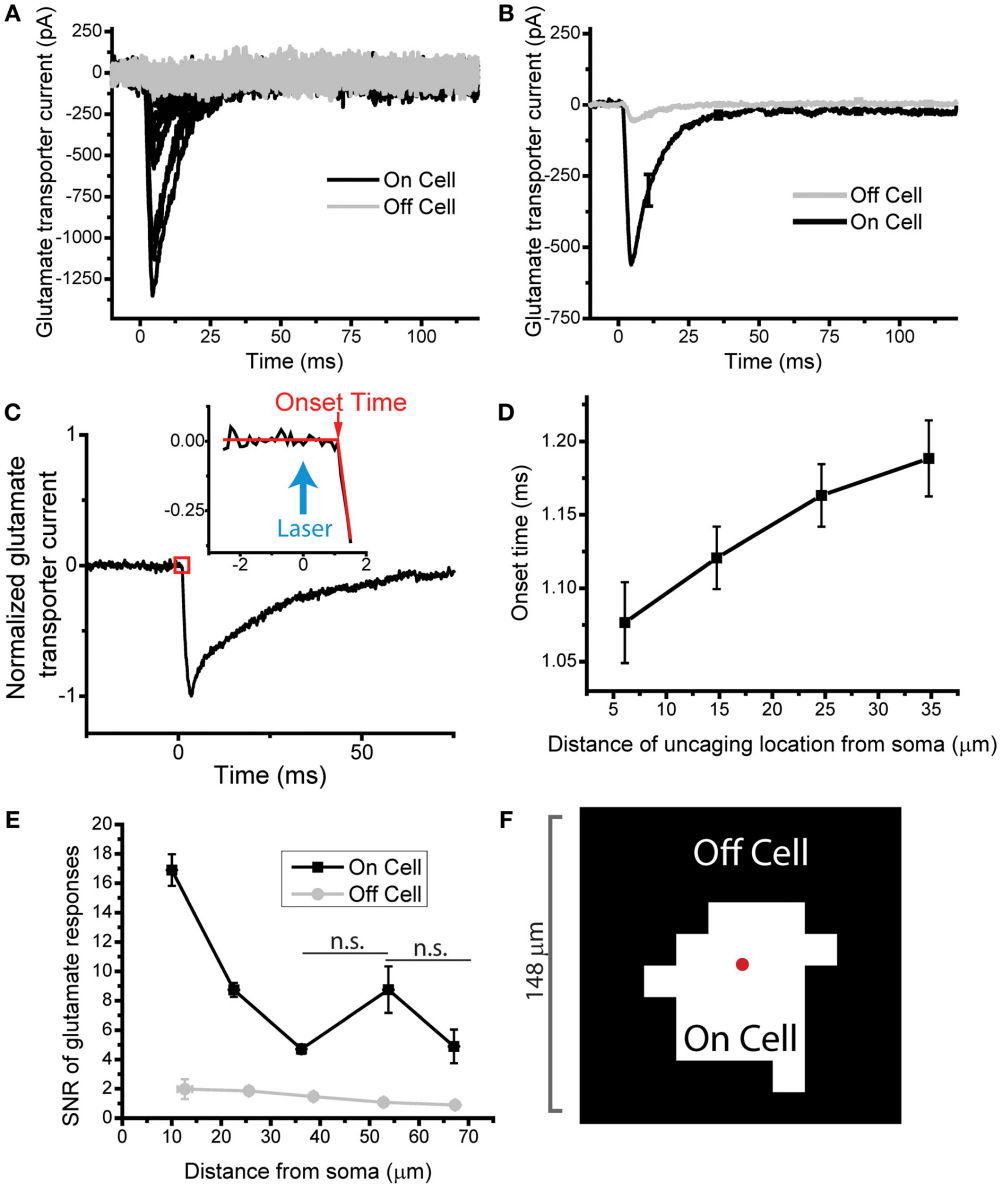
**Identifying the glutamate responsive area for individual astrocytes. (A)** a representative astrocyte (P29 Sham), showing a subset of glutamate evoked transporter currents segregated into the On-domain (black) and Off-domain (gray) responses. Locations were grouped area based on onset time of the glutamate current (see methods). Each trace is an average of 3 uncaging trails on the same uncaging location. 55 traces were On-domain and 45 traces were Off-domain. **(B)** Average On-cell and Off-domain responses of the cell pictured in A. Off-domain average shows small glutamate evoked currents with a delayed onset time suggesting indirect activation of glutamate transporters. **(C)** An example glutamate uncaging trace with a piecewise function fit to estimate the onset time of the glutamate evoked current. **(D)** Onset times scale with distance between the uncaging spot and the soma. *N* = 33 cells, P26–34 Sham, with 850 uncaging traces overall. **(E)** Signal to noise ratio of glutamate current across distance from the soma, for On and Off cell locations, demonstrating that the On-domain currents remain above the noise. *N* = 33 cells, P26–34 Sham cells. **(F)** Example glutamate responsive map of a mature sham astrocyte. 22 responsive spots out of 100 total spots. Average spacing 16.4 μm between spots, red dot marks approximate soma location.

Using a steerable photoactivation laser allows photolysis of glutamate at different spatial locations relative to the cell body of the patched astrocyte. In order to systematically map the glutamate responsive area, the area from which the astrocyte clears glutamate, 100 uncaging locations in a 10 × 10 grid with an average distance of 16.4 ± 0.2 μm between uncaging spots were utilized (example map Figure 2F). Our LSAM approach used single-photon photoactivation of MNI-glutamate; while this technique lacks the resolution to map synaptic microdomains of glutamate clearance, it generated large TCs, crucial to spatially mapping TCs in low resistance astrocytes. The spatial resolution we achieved was similar to other single photon photolysis strategies (Katagiri et al., 2007; Purgert et al., 2014).

### Identifying the glutamate-responsive domain of a single astrocyte

We next implemented a system to categorize photolysis locations as On-domain (glutamate responsive) or Off-domain (not glutamate-responsive) (Figure 1D). The main parameter of evoked TCs that provided information regarding the glutamate responsiveness of a given photolysis location was the current onset time (see methods, Figures 2A,B). The TC onset time was quantified by an automated algorithm used to fit a piecewise function to the initial 5 ms of the evoked TC at a given photolysis location (Figure 2C). Locations which could not be fit with high statistical confidence were automatically categorized as Off-domain. This created a signal to noise filter independent of the additional temporal filter.

Once the onset time was determined, two temporal criteria were used to further delineate between On- and Off-domain. First, a lower limit of 0.75 ms from the center of the uncaging pulse to the detected onset time was used to filter aberrant signals. From somatic uncaging onto a large number of cells (*N* = 72), we found that there was a minimum of 0.75 ms delay between the center of the uncaging pulse and the onset of the evoked current. This delay can be attributed to uncaging time, transporter binding, and the act of transport. Second, an upper limit of 2.25 ms from the center of the uncaging pulse to the time of TC onset was used. This puts a limit of 1.5 ms diffusion time from the site of uncaging to a process on the recorded astrocyte. These limits were based on known biophysical parameters (Rusakov and Kullmann, 1998; Grewer et al., 2000; Canepari et al., 2001; Kessler, 2013) and our own data which drove the development of these criteria.

To test our criteria we used two simple assays. First, based on cable properties and signal propagation, it is predicted that the onset of the evoked current should increase with the distance from the soma. Our results are consistent with this prediction (Figure 2D) but the amount of delay in the onset time even at locations greater than 30 μm from the astrocyte soma is much smaller than the upper limit of 2.25 ms. Second, we examined the signal-to-noise ratio (SNR) of TCs categorized as On- and Off- domain. The onset time criteria used do not set an explicit signal to noise threshold; therefore we measured the signal to noise of the On-domain responses to provide an independent readout of the characterization. The SNR amplitude does follow a distance-dependent drop off, as would be predicted when making recordings in low membrane resistance astrocytes, but On-domain responses remain well above the noise of the signal even out into the distal processes of the astrocyte (Figure 2E). The increase in SNR around 55 μm from the soma is not statistically different from its neighboring points, two sample *t*-test *p* > 0.25, 0.08. Together, these tests show our ability to detect TCs at distal locations, and confirms that even when TCs are filtered by the astrocyte membrane properties, On- vs. Off-domain responses can be resolved. An example On- vs. Off- cell map, created using these criteria is shown in Figure 2F.

### Ensuring the validity of mapped receptive fields

In order to further validate this novel approach and to ensure the specificity, reproducibility, and sensitivity of the evoked currents, a number of controls were performed. First, we utilized a high density line scan photolysis protocol. Here, a single line across the patched astrocyte was photoactived at 5 μm intervals, ensuring spatial over-sampling of the glutamate-receptive field given our spot size of 10 μm. This approach did not reveal new spatial features and almost identically replicated grid mapped receptive fields, suggesting that we are not under-sampling based on our photolysis resolution (data not shown). It should be noted that the astrocyte morphology itself is not oversampled; LSAM is limited by the photolysis spatial resolution and the ability to resolve evoked signals in low-resistance cells.

**Figure 3:**
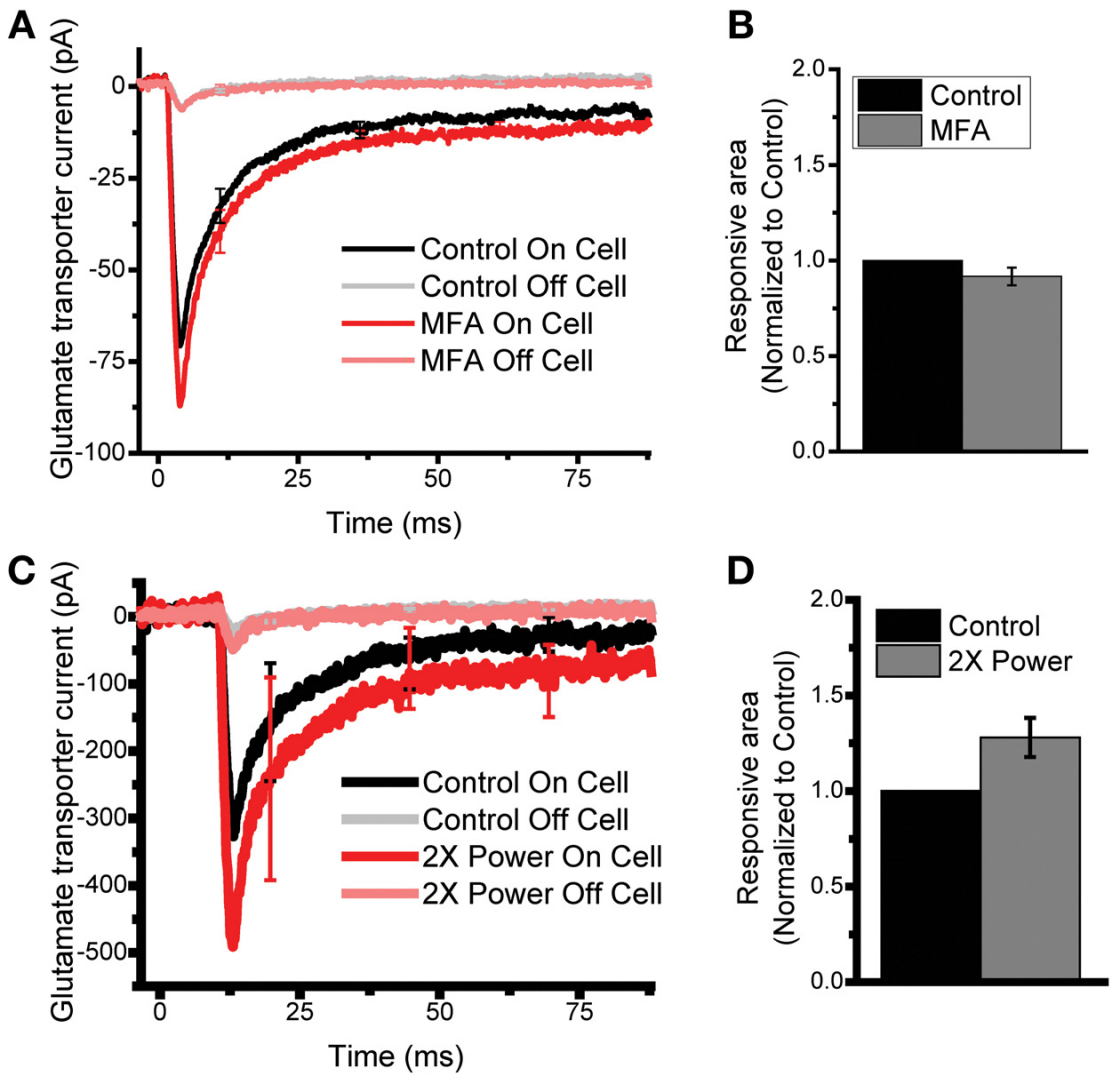
**Characterizing the specificity of spatial glutamate uncaging. (A)** In order to control for possible glutamate evoked current coming from gap junction coupled neighboring cells, we compared On and Off cell responses before (black and gray) and after the application of the gap junction inhibitor MFA (100 μM, red and pink). No difference is seen in On or Off cell traces after application of MFA. *N* = 10 cells. **(B)** For the cells in A, the glutamate responsive area was quantified. No significant difference was seen following the application of MFA, paired *t*-test *p* > 0.6. **(C)** In order to control for the glutamate sensitivity of the assay, we mapped cells at normal (black and gray) and 2X normal laser uncaging power (red and pink). *N* = 6 cells. **(D)** For the cells in C, the glutamate responsive area was quantified, no significant difference is seen, paired *t*-test *p* > 0.07.

Next, we examined the possibility that the TCs recorded in a patch-clamped astrocyte are actually generated in a neighboring astrocyte and spread passively via gap-junction coupling. To address this, astrocyte receptive field maps were generated before and after application of the gap junction inhibitor meclofenamic acid (MFA, 100 μM) (Xu et al., 2010). Inhibiting the gap junctions significantly increased the membrane resistance 4.3 ± 0.4 MΩ, 8.1 ± 0.8 MΩ for control and MFA respectively agreeing with previous results (Meme et al., 2009) (log corrected paired *t*-test *p* < 0.0003, *N* = 7 cells). Application of MFA did not alter on-domain traces (On-domain Integrated current 1.06 ± 0.21 MFA normalized to control *p* > 0.8 paired *t*-tests, On-domain T_1/2_ decays 5.0 ± 0.5 ms and 4.2 ± 0.6 ms for control and MFA respectively *p* > 0.27, *N* = 7 cells, Figure 3A). Importantly, the glutamate responsive area was unchanged by the MFA block of gap junctions (MFA area 0.92 ± 0.05, normalized to control, Figure 3B). Although there is a trend toward a slight decrease in responsive area, this change is not significant. This data shows that TCs are not generated by transporters in gap junction-coupled neighboring cells nor does the spread of current through gap junctions broadened glutamate receptive fields assayed via LSAM.

Lastly, the amount of glutamate uncaged could be limiting the sensitivity of the assay. In order to test if glutamate transporter currents and astrocyte receptive fields are sensitive to the amount of glutamate uncaged, we took measurements of receptive fields in the same cell at standard laser power, and at double the standard laser power. If the sensitivity of the assay is limited by the amount of glutamate uncaged we should see a larger glutamate responsive area with increased laser power. While we did see an increase in the integrated glutamate current (1.77 ± 0.16, 2X power normalized to control, *p* < 0.003 log corrected paired *t*-test *N* = 5 cells, Figure 3C); we saw no significant change in the glutamate responsive area when the laser power was doubled (glutamate responsive area at 2X laser power normalized to control 1.3 ± 0.1, Figure 3D). There is a trend toward increased glutamate photolysis leading to increased map size, but again, this change is not statistically significant. These controls show that glutamate responsive areas are largely insensitive to changes in glutamate concentrations or changes in gap junction coupling. Together, these findings demonstrate that LSAM provides a reliable method of measuring the glutamate responsive domain of an individual astrocyte.

### LSAM reveals developmental and FL-induced changes in astrocyte glutamate responsive domains

**Figure 4:**
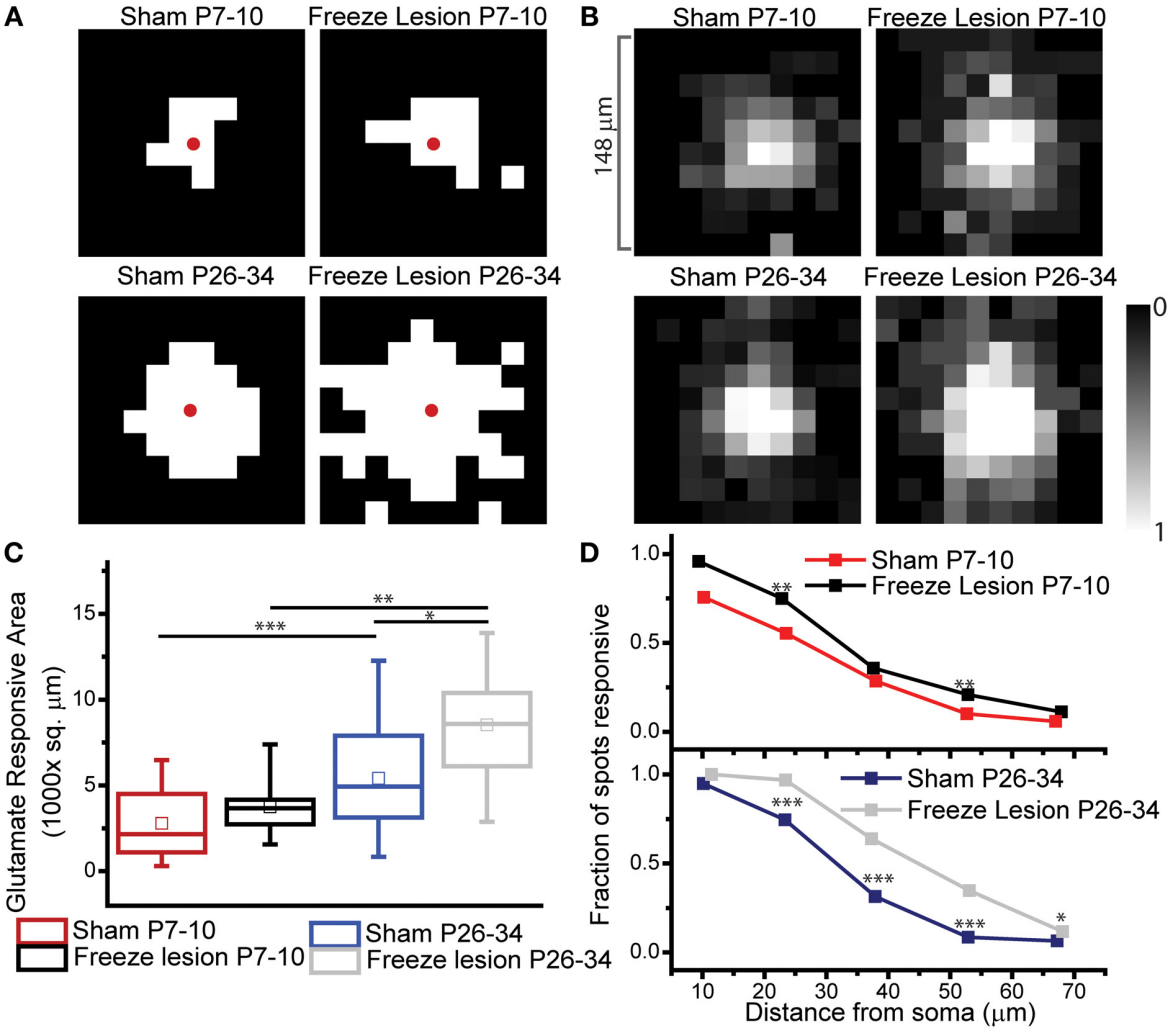
**Mapping glutamate responsive domains**. A 10 × 10 grid of uncaging locations around the patched astrocyte, with 16.4 μm spacing between locations and a 10um spot size enables the characterization of the glutamate responsive domain of individual astrocytes. **(A)** Single cell example maps of glutamate responsive domains. White squares represent an On-domain response at the given location, a Black square represents an Off-domain response based upon the glutamate evoked current. The red dot marks approximate soma location. **(B)** Average glutamate response maps. Grayscale from White (all cells responsive) to Black (no cells responsive). **(C)** Quantification of the glutamate responsive area pictured in B. Box and whisker plots, whiskers (min/max), box 25, 50, 75% quartiles, and square (mean). Astrocytes increase their glutamate responsive domain through development, and FL P26–34 astrocytes show a significantly increased glutamate responsive area compared to sham. One way log corrected Anova, Bonferroni test. Sham P7–10 vs. FL P7–10 *p* > 0.3, Sham P7–10 vs. Sham P26–34 *p* < 0.0007, FL P7–10 vs. FL P26–34 *p* < 0.008, FLP26–34 vs. Sham P26–34 *p* < 0.02. **(D)** The fraction of uncaging spots that are responsive (On-domain) based on the distance from the soma for all conditions. 24–300 uncaging spots per condition, *N* = 13, 9, 36, 19 cells for Sham P7–10, FL P7–10, Sham P26–34, and FL P26–34 respectively. Sham vs. FL P7–10 *p* < 0.008 (15–30 μm), *p* < 0.004 (45–60 μm) Sham vs. FL P26–34 *p* < 0.0001 (15–30, 30–45, 45–60 μm), *p* < 0.03 (60–75 μm), Fisher exact test. ^*^p < 0.05, ^**^p < 0.01, ^***^p < 0.001 tests as described.

Using the LSAM approach described above, we mapped the glutamate responsive domains from sham and FL astrocytes at neonatal (P7–10) and mature (P26–34) timepoints (note: not all developmental processes are truly matured by P26–34, but for the sake of simplicity the term mature will be used from here on). The glutamate responsive area of each individual astrocyte was mapped (Figure 4A). These individual astrocyte maps are then aligned based on soma location and averaged to create a density map of glutamate responsive area (Figure 4B). During development from neonatal to mature astrocytes in sham treated animals, there was a significant increase in the average astrocyte domain (glutamate responsive area: neonatal 2788 ± 604 μm^2^ and mature 5415 ± 493 μm^2^ *N* = 13, 36 cells respectively). Interestingly, mature astrocytes following FL show an even greater increase in glutamate responsive area compared to mature sham astrocytes (glutamate responsive area: neonatal FL 3754 ± 569 μm^2^, mature FL 8529 ± 695 μm^2^, *N* = 9, and 19 cells respectively. Log corrected one way Anova, Bonferroni test. Sham P7–10 vs. Sham P26–34 *p* < 0.0007, FL vs. Sham P26–34 *p* < 0.02, FL P7–10 vs. P26–34 *p* < 0.008, Figure 4C). When we quantified the fraction of photolysis locations that are responsive to glutamate uncaging at a given distance from the soma, the results agree with the area mapping and reveal subtle, but significant changes in the distribution of neonatal FL responsive locations that were not revealed by the area mapping (Figure 4D). We found that in astrocytes in the FL neonatal cortex there were more responsive locations close to the soma and as well as distally, although changes were not present at all distances. In the mature FL astrocytes, the fraction of responsive spots is significantly increased across the entire span of the astrocyte, in line with our finding of increased area.

**Figure 5:**
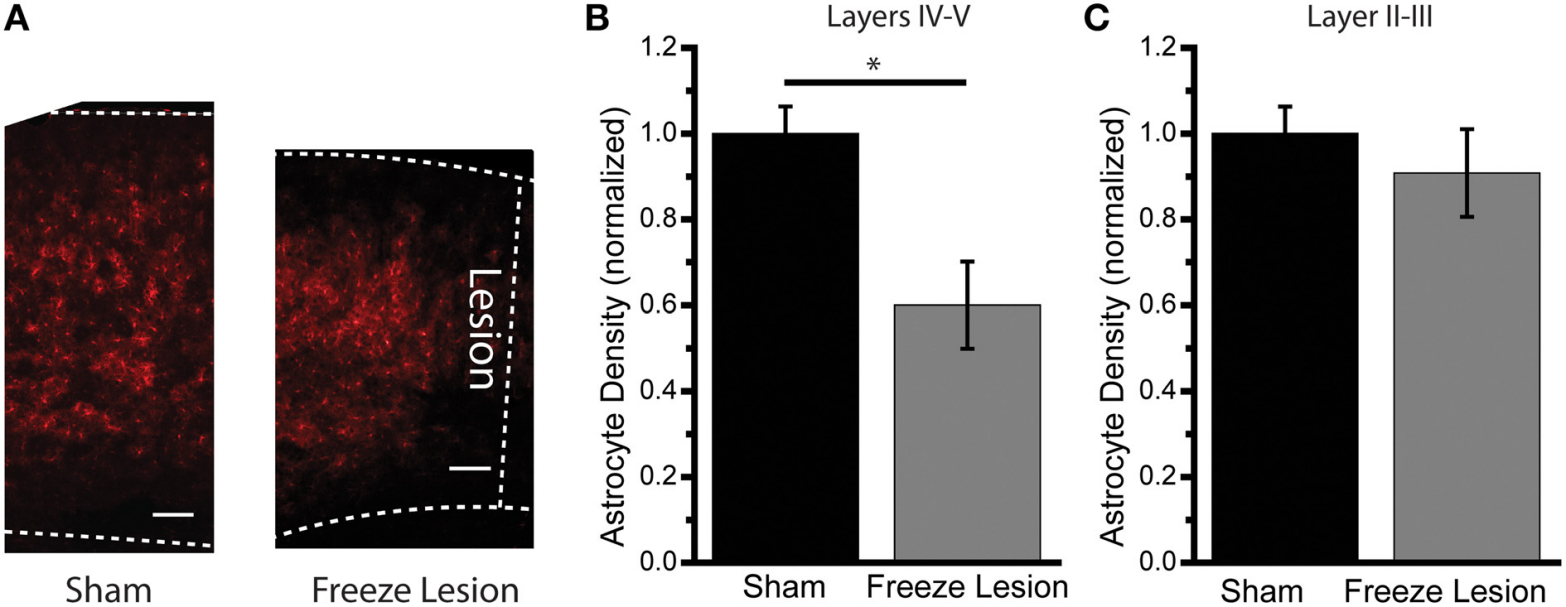
**Astrocyte density following freeze lesion**. Using human EAAT2 promoter driven tdTomato reporter mice **(A)**, the number of astrocyte cell bodies in the paramicrogyral zone adjacent to the lesion in layers IV–V **(B)** and Layers II–III **(C)** were quantified in P27–29 Sham and FL mice. Deep layers showed a significant decrease in astrocyte density *p* < 0.03, while layers II–III showed no significant difference in astrocyte density *p* > 0.5, two sample *t*-test *N* = 3 Sham, *N* = 5 FL mice Scale bar 100 μm. ^*^p < 0.05 *t*-test.

Using reporter mice in which astrocytes express the tdTomato fluorophore under control of the human EAAT2 promoter (Yang et al., 2011), we examined changes in the astrocyte density following FL at P27–29 (Figure 5A). Counting labeled cell bodies in deep (layers IV–VI) (Figure 5B) and superficial (layers II–III) (Figure 5C) areas of the paramicrogyral zone adjacent to the site of the FL showed a specific reduction in the number of astroctyes in the deeper layers of the cortex following FL. This confirms previous findings in the rat model of FL (Dulla et al., 2012).

### Glutamate transporter currents are not uniform within single astrocytes

**Figure 6:**
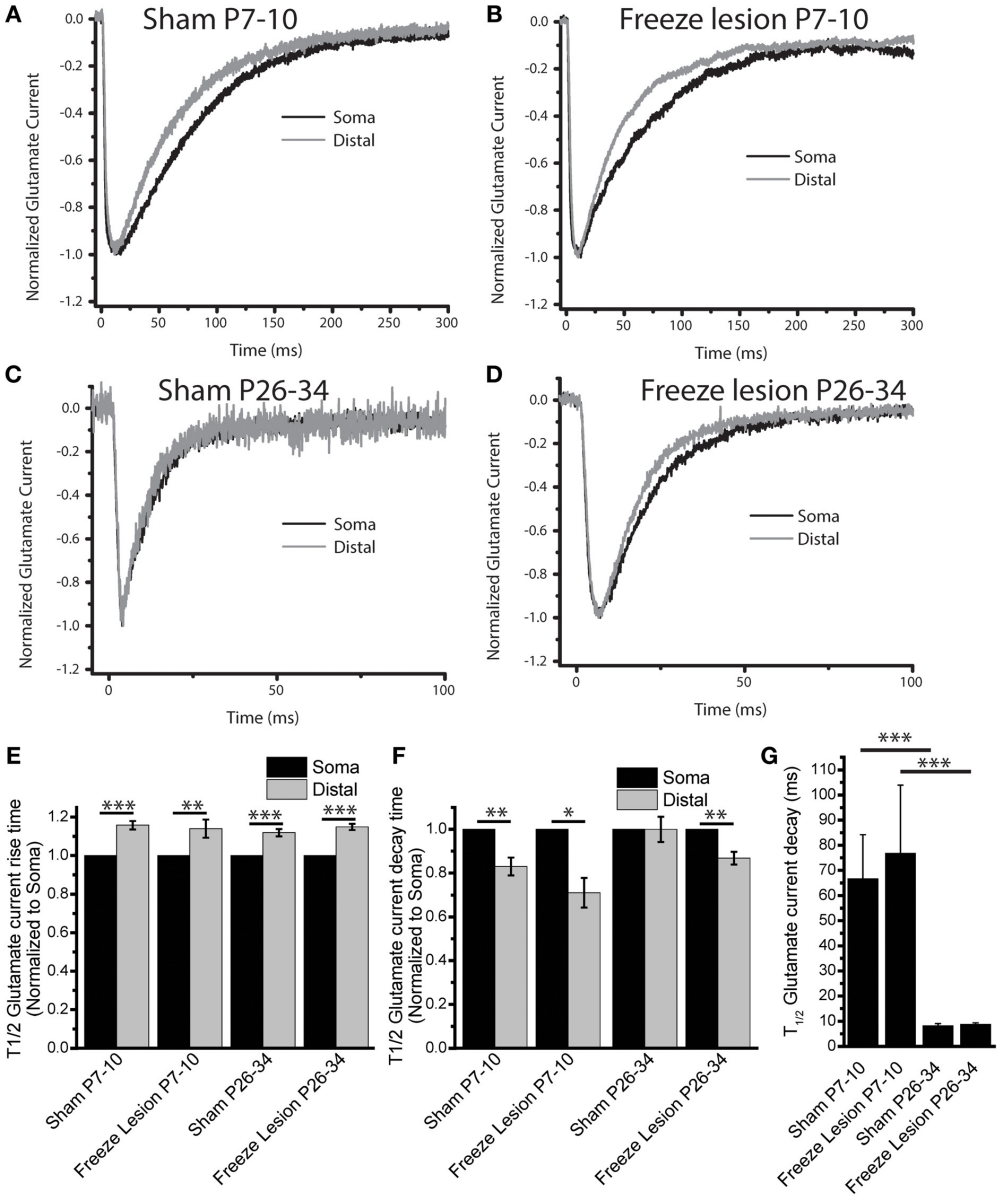
**Kinetic changes between somatic and distal glutamate clearance**. Example traces for Somatic (Black) and Distal (Gray) glutamate evoked transporter currents, normalized to peak, for **(A)** Sham P7–10, **(B)** Freeze Lesion P7–10, **(C)** Sham P26–34, and **(D)** Freeze Lesion P26–34 **(E)** The rise-time of the transporter current is significantly slowed in all conditions, paired *t*-test *p* < 0.0003, *p* < 0.007, *p* < 0.0003, and *p* < 0.000003, and *N* = 11, 9, 26, and 18 cells for Sham P7–10, Freeze Lesion P7–10, Sham P26–34, and Freeze Lesion P26–34. **(F)** T_1/2_ of evoked glutamate current decay is representative of glutamate clearance. Distal (uncaging location >20 um from soma) transporter currents show faster decay than somatic currents except for Sham P26–34, which showed no significant difference. Log corrected paired *t*-test *p* < 0.002, *p* < 0.02, *p* > 0.25, *p* < 0.006 for Sham P7–10, Freeze Lesion P7–10, Sham P26–34, and Freeze Lesion P26–34, respectively. **(G)** Changes in currents through development. T_1/2_ of glutamate current decay kinetics show no difference between Sham and Freeze lesion at neonatal timepoints and mature timepoints, but are accelerated through development. Sham P7–10 vs. P26–34, *p* < 3^*^ 10^−11^; FL P7–10 vs. P26–34 *p* < 2^*^ 10^−7^ log corrected ANOVA Bonferroni test. ^*^p < 0.05, ^**^p < 0.01, ^***^p < 0.001 tests as described.

LSAM not only allows mapping the presence or absence of glutamate transporter currents at different spatial locations, but also permits measurement of differences in TC kinetics within individual astrocytes. Having categorized On-domain responses, we further binned the On-domain responses into somatic (<20 μm from the center of the soma) and distal (>20 μm from the soma) in order to compare the kinetics of extracellular glutamate between different cellular locations and between different conditions. At somatic locations we see an acceleration of the decay kinetics as animals mature. This was seen in both sham (66.6 ± 11.7 and 8.2 ± 9.1 ms for neonatal and mature, *N* = 11, 26 cells respectively *p* < 3^*^ 10^−11^, log corrected ANOVA Bonferroni test) and FL astrocytes (76.8 ± 27.2 ms and 8.8 ± 5.9 ms for neonatal and mature *N* = 9, 18 cells respectively, *p* < 2^*^ 10^−7^, log corrected ANOVA Bonferroni test Figure 6G). This agrees with previous results showing an acceleration of glutamate clearance during development (Diamond, 2005). Surprisingly, we did not find differences in the kinetics of TCs between sham and FL astrocytes at either time-point using this assay (*p* > 0.7 neonatal and *p* > 0.2 mature, log-corrected two-sample *t*-test).

Next we characterized changes in TC decay kinetics at distal vs. somatic locations within individual cells (Figures 6A–D). Astrocytes in the neonatal sham and FL cortex showed a small, but highly significant acceleration of the TC decay time at distal locations compared to the soma (distal T_1/2_ time constants of glutamate evoked current normalized to decay kinetics at somatic locations: neonatal sham 0.83 ± 0.04, neonatal FL 0.71 ± 0.07). These spatial distinctions in TC decay time were absent in mature astrocytes in the sham lesioned animals, with TC decay times being almost identical at the soma and more distal locations. Interestingly, astrocytes in the FL mature cortex continued to have faster TC kinetics at distal processes than at the soma (distal T_1/2_ time constants of glutamate evoked current normalized to decay kinetics at somatic photolysis locations: mature sham 1.00 ± 0.06, neonatal FL 0.87 ± 0.003, Figure 6F).

In interpreting this data care must be taken as transporter currents are filtered based on the membrane properties and propagation distance. Based on the delayed onset time and capacitive filtering of the propagation of the TC from distal locations, we would expect that distal currents should be slowed compared to the somatic currents. The rise-time of the glutamate evoked current was significantly slowed for distal compared to somatic current under all experimental conditions (T_1/2_ rise-times of glutamate evoked current at distal photolysis locations normalized to somatic locations: 1.16 ± 0.02, 1.14 ± 0.54, 1.12 ± 0.02 1.15 ± 0.02 for neonatal sham, neonatal FL, mature sham, and mature FL respectively Figure 6E), as expected. These data indicate that although electrotonic filtering of TC occurs, it alone is unlikely to explain changes in TC decay kinetics at proximal vs. distal locations. Together, these findings show that TC kinetics are slightly, but significantly accelerated in the distal processes of cortical astrocytes both early in development and in mature astrocytes following neonatal FL injury.

### Freeze lesion alters the maturation of GLT-1 vs. GLAST functional contribution to astrocyte glutamate uptake

Astrocytes utilize the glutamate transporters GLT-1 and GLAST to clear glutamate from the extracellular space, with GLAST being dominant at neonatal time points and GLT-1 being the dominant transporter at mature time points (Furuta et al., 1997). In order to test if the functional balance of transporters is disrupted following FL, we examined the effects of the GLT-1 specific inhibitor DHK (Arriza et al., 1994) (300 μM) on integrated TC and centroid of the TCs. Integrating TCs provides information about how much total glutamate is transported into the recorded astrocyte. The centroid of the TC is used to characterize astrocytic glutamate transport function (Diamond, 2005; Scimemi et al., 2009; Thomas et al., 2011). If there is robust functional transport, glutamate will be moved into the cell rapidly and the TC centroid will occur early. On the other hand, if transport is less abundant, glutamate will be moved in more slowly and the TC centroid will occur later. Using these quantitative tools in combination with DHK we probed the developmental maturation of TCs in the sham and FL cortex at somatic locations.

**Figure 7:**
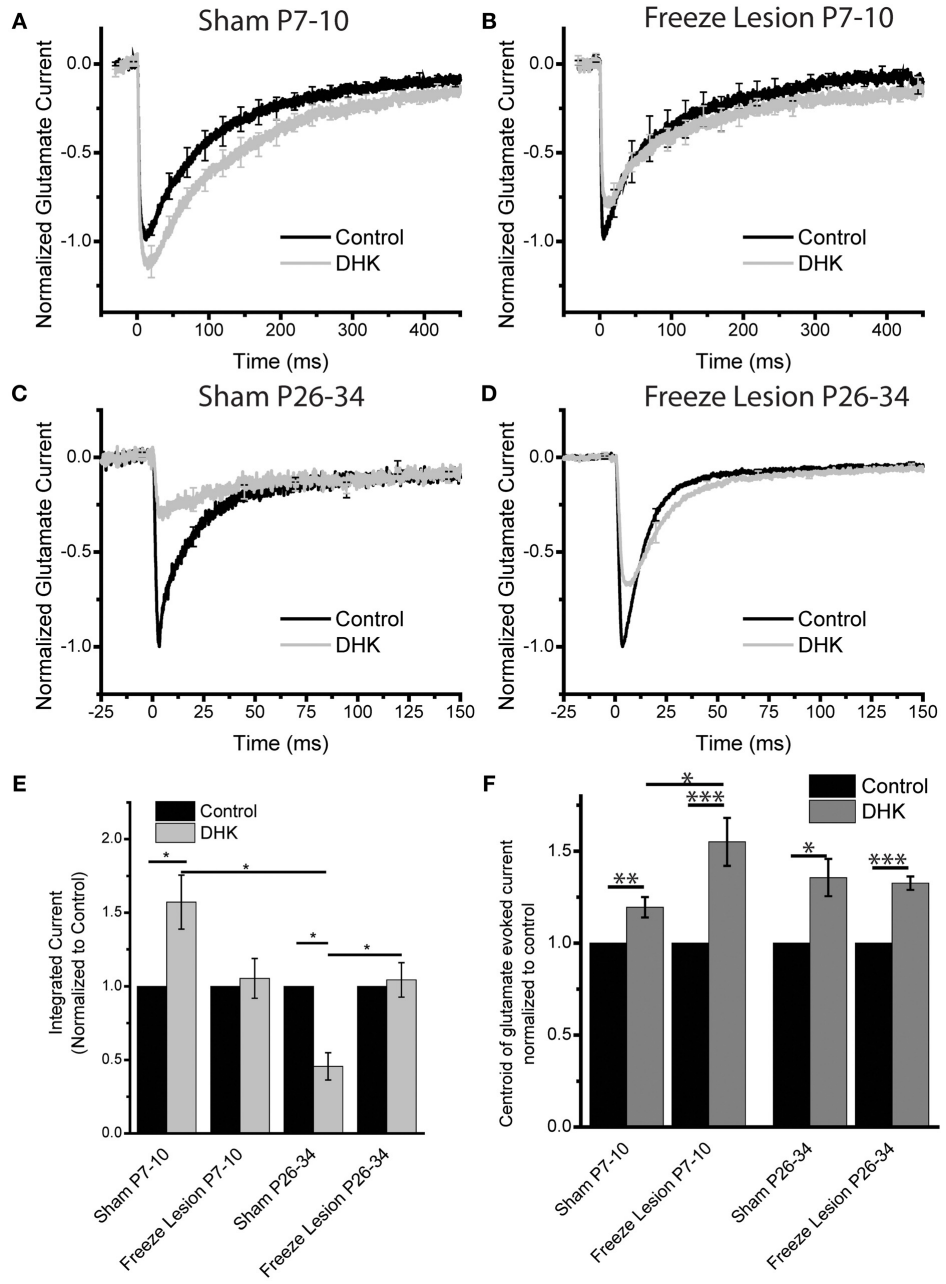
**Freeze lesion alters EAAT subtype-specific functional maturation in astrocytes**. Somatic glutamate evoked traces are recorded before and after the application of the GLT-1 inhibitor DHK (300 μM), for **(A)** Sham P7–10, **(B)** Freeze Lesion P7–10, **(C)** Sham P26–34, and **(D)** Freeze Lesion P26–34. Average of *N* = 10, 8, 5, and 10 cells respectively. **(E)** Integrated glutamate evoked currents, normalized to control, shows a significant increase following DHK application in Sham P7–10 astrocytes *p* < 0.02. Sham P26–34 shows a significant decrease in integrated current *p* < 0.02. The effects of DHK on integrated TC current are significantly different between neonatal and mature sham astrocytes and between mature FL and sham astrocytes. log corrected ANOVA Bonferroni test **(F)** Centroids of glutamate evoked currents of P7–10 and P26–34 Sham and Freeze lesion show slowing of glutamate reuptake following DHK application. P7–10 Sham *p* < 0.007, P7–10 Freeze lesion *p* < 0.0006, P26–34 Sham *p* < 0.02, P26–34 Freeze Lesion *p* < 4^*^ 10^−6^ log corrected paired *t*-test. P7–10 Freeze lesion astrocytes show an enhanced slowing compared to P7–10 sham following DHK application *p* < 0.02 two sample *t*-test. ^*^p < 0.05, ^**^p < 0.01, ^***^p < 0.001 tests as described.

In neonatal sham astrocytes, DHK significantly shifted the centroid of the TC for neonatal sham and FL astrocytes slowing the kinetics (Figures 7A,F). The effect of DHK on the TC centroid in neonatal FL astrocytes, was significantly larger compared to sham (Figure 7F). The integrated TC was significantly increased by application of DHK. This effect was not present in FL neonatal astrocytes (integrated glutamate evoked current after DHK normalized to control sham = 1.6 ± 0.2, *p* < 0.02, *N* = 10; FL = 1.05 ± 0.1 *p* > 0.36 *N* = 8, log corrected ANOVA Bonferroni test, Figures 7A,E). This suggests that there is a greater dependence on GLT-1 for astrocytic clearance of glutamate in neonatal astrocytes following FL.

We next examined astrocytes in the mature cortex and found that neonatal FL induced long-term changes in glutamate transport. Mature astrocytes in the sham lesioned cortex were heavily reliant on GLT-1, as shown by their high sensitivity to DHK (Figure 7C). With GLT-1 blocked, the integrated current evoked by photolysis of MNI-glutamate was significantly reduced. This suggests that without GLT-1, mature sham-lesioned astrocytes are largely incapable of removing extracellular glutamate in an equivalent time-frame. In astrocytes in the FL cortex, abundant non-GLT-1 mediated uptake was present. The integrated TC was unchanged and a robust a TC was seen in the presence of DHK (Figure 7D). This indicates that non-GLT-1 uptake mechanisms, are capable of transporting extracellular glutamate in astrocytes in the mature FL cortex (Figure 7E). The centroid of the TC is slowed following DHK for both mature sham and FL astrocytes. Surprisingly it showed no difference between the sham and FL. Following FL, astrocytes appear to rely more on GLT-1 at early developmental time points (Figure 7B) and less on GLT-1 at mature time points (Figure 7D). Distal photolysis locations showed similar results to the somatic locations for all conditions (data not shown). Together these results are consistent with our hypothesis that neonatal lesion disrupts the maturation of astrocytic glutamate transport.

## Discussion

Here we developed a novel technique, laser-scanning astrocyte mapping (LSAM), and have used it to investigate changes in astrocytes in the FL model of developmental cortical malformation. While previous studies have utilized anatomical and immunohistochemical approaches, there has been little success at understanding the functional distribution of glutamate transport within astrocytes. We developed LSAM to help answer some of these previously unexamined questions and to test the hypothesis that neonatal cortical freeze lesion disrupts the normal maturation of functional glutamate uptake. This novel approach showed that astrocytes in the mature FL have an increased glutamate responsive area. Second, astrocytes in the adult sham vs. FL cortex have a significant alteration in distal vs somatic TC decay kinetics. Lastly, TCs in the adult FL cortex are surprisingly DHK-insensitive, suggesting another transporter, such as GLAST, may carry a large amount of the TC. The functional properties of neonatal astrocytes remained surprisingly intact following FL, although LSAM revealed significant changes in the glutamate-responsiveness of astrocytes at specific locations. By combining LSAM with a model of developmental lesion, we have been able to begin to parse the functional, and specifically spatial, maturation of astrocytic glutamate uptake in the cerebral cortex.

Previously, astrocytes were probed with anatomical techniques (Kosaka and Hama, 1986; Zhuo et al., 1997; Bushong et al., 2002; Ogata and Kosaka, 2002; Oberheim et al., 2008, 2012). These approaches revealed that astrocyte morphological maturation is characterized by an expansion of astrocyte processes and transformation from long, filipodial-like processes to a highly branched, fusiform structure (Bushong et al., 2004; Morel et al., 2014). Our results are consistent with and complement these studies. We report that glutamate-responsive domain size increases during development which likely reflects changes in astrocyte morphology and large scale increases in EAAT expression. Using LSAM, we also have been able to determine that there is developmental shift in functional glutamate uptake within individual cells. In the neonatal cortex, astrocytes have faster TC decay times in their distal processes as compared to at the soma. This suggests a shift in transporter density, that when EAAT expression is low, as it is in the neonatal cortex, glutamate transport function is likely targeted to the growing tips of astrocyte processes. GLT-1 is known to be preferentially localized to peri-synaptic regions of astrocytes (Rothstein et al., 1994; Chaudhry et al., 1995). In the neonatal cortex, there may be an increased need for active transport at synaptic sites where distal processes are likely located. As astrocytes mature and ramify, and overall transporter levels increase, there is likely a more homogeneous distribution of transporters which equalizes distal and somatic TC decay times.

Using LSAM in the mature FL cortex, we found that astrocytes have an increased average glutamate responsive area, as compared to sham cortex. These findings are consistent with findings in both chemo-convulsant and genetic models of epilepsy in which astrocytes increase their domain size. In these studies, astrocytes lose their tiled, non-overlapping domains and neighboring astrocytes have intermingled processes (Oberheim et al., 2008). Whether this occurs in the FL is unknown. Additionally we have shown a decrease in astrocyte density in the deep layers of cortex following FL, replicating previous findings in rats (Dulla et al., 2012). Perhaps astrocytes compensate for a decrease in their numbers with an increase in their glutamate responsive area. The functional implications of this change are not yet clear, but if each astrocyte maintains the glutamate environment from a larger spatial domain, glutamate uptake may be more easily compromised or metabolic recycling of neurotransmitter precursors may be less efficient (Tani et al., 2014). Future studies using paired astrocyte recording and LSAM, and more traditional anatomical approaches, will attempt to delineate between these two possibilities. Additionally we report that in the adult FL cortex, TC decay kinetics are faster at distal vs. somatic locations. This could be due to an increase in distal surface transporter density, to more directly couple with synapses in the hyperexcitable cortex. Previous studies have demonstrated an increase in mEPSPs in the paramicrogyral zone following FL (Jacobs and Prince, 2005; Bell and Jacobs, 2014), suggesting there may be an increased demand for glutamate uptake.

Examining TCs with the GLT-1 specific inhibitor DHK enabled quantification of GLT-1 vs. non-GLT-1 transport throughout development and following FL. DHK caused a modest change in TC kinetics in astrocytes in sham injured neonatal cortex, consistent with largely non-GLT-1-mediated (likely GLAST-mediated) transport. Following FL, however, TC kinetics were prolonged more by DHK application than in the sham lesioned cortex. This suggests that there is more reliance on GLT-1 at neonatal time points in the FL cortex. Through development, in sham-injured animals, astrocytes TCs shift from being relatively DHK-insensitive to highly DHK-sensitive. GLT-1 is known to be the dominant astrocytic glutamate transporter at mature time points (Furuta et al., 1997), in line with our findings. While slowing of the centroids in the mature condition are not significantly different, given the very low levels of available transporters in the Sham-DHK condition, it is unclear how accurately the currents still report the extracellular glutamate concentrations. The question as to how glutamate gets cleared from the extracellular space in the Sham-DHK condition remains. Glutamate could still be taken up by astrocytes very slowly, which would be difficult to resolve; alternatively the glutamate could escape into the perfusate when GLT-1 is blocked. Mature astrocytes following FL do not show an effect on the integrated current in the presence of DHK, which suggests a non-GLT-1 mediated transport compensates for the pharmacologically blocked GLT-1. This shows a shift in the developmental profile of how glutamate is cleared following the FL injury. The implications of the changes in GLT-1 function following FL are not clear, but suggest further investigation is required into the distinct role of each transporter in regulating extracellular glutamate.

**Figure 8:**
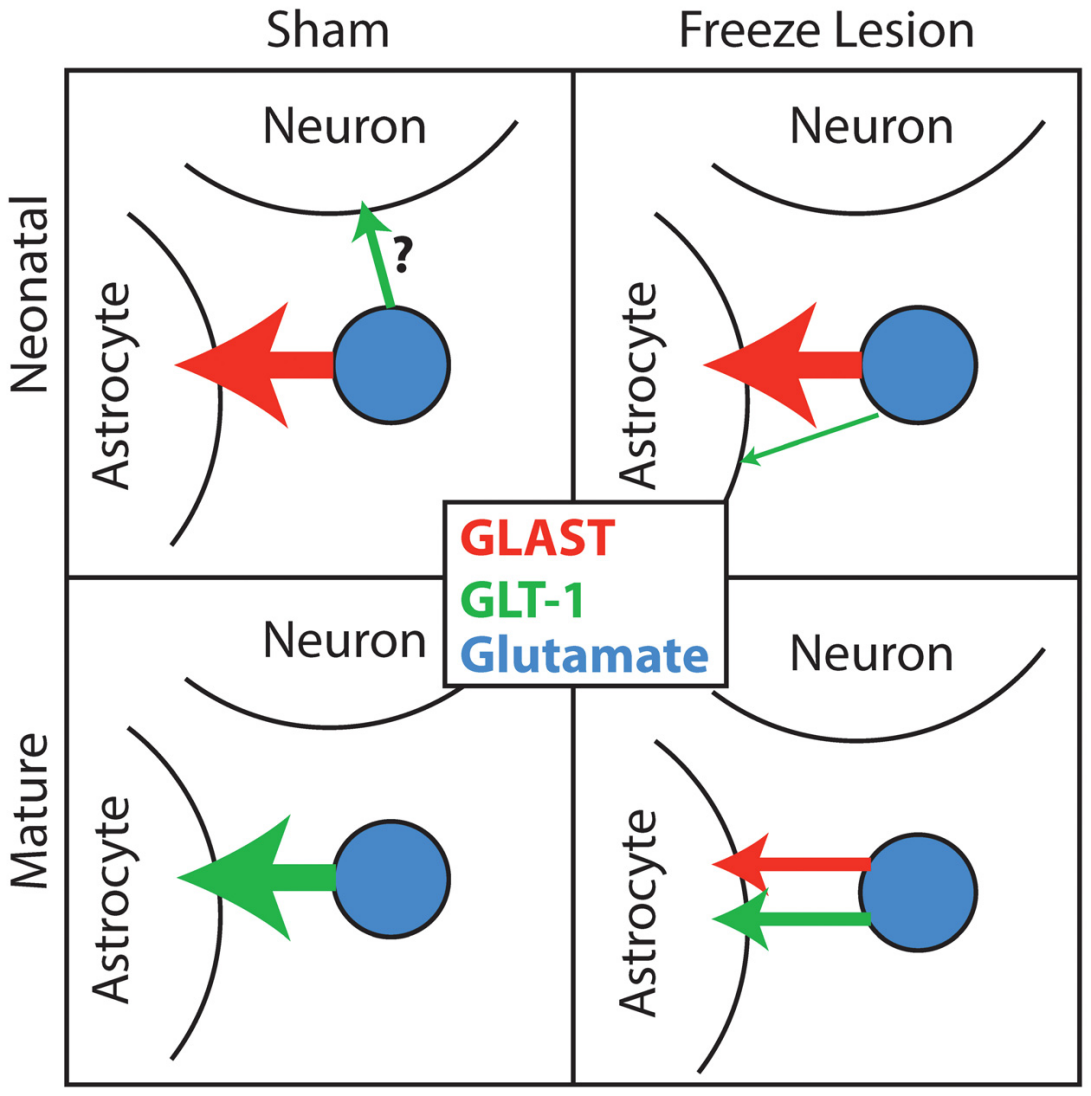
**Glutamate transporter utilization through development**. Based on our findings, we postulate the following mechanism for the utilization of EAATs in clearing uncaged glutamate. In the sham-lesioned condition, neonatal uptake is largely mediated by GLAST in astrocytes with some possible contribution by neuronal (or other non-astrocytic) GLT-1. As astrocytes mature, they rely heavily on GLT-1 instead of GLAST. In the FL cortex, neonatal astrocytes utilize more GLT-1-dependent transport. In the mature FL cortex, astrocytes use robust GLT-1 and GLAST function to remove glutamate.

Surprisingly, DHK increased the integrated TC in neonatal sham astrocytes. This suggests that there is potential heterogeneity within astrocytes and/or non-astrocytic DHK-sensitive transporter expression (see review Danbolt, 2001) (Brooks-Kayal et al., 1998; Mennerick et al., 1998; Chen et al., 2002; Schmitt et al., 2002; Chen and Swanson, 2003; Scimemi et al., 2009; DeSilva et al., 2012). While this heterogeneity in glutamate sinks could be present in other conditions, only the very slow glutamate clearance kinetics and lack of astrocytic GLT-1 in the neonatal sham astrocytes may enable detection of these glutamate sinks specifically in this condition (Figure 8). The increased reliance on GLT-1 in astrocytes in the neonatal FL cortex, is demonstrated by the larger change in TC centroid upon application of DHK.

There are a number of limitations to our studies. First, astrocytes are a low-resistance cell type making it difficult to measure currents evoked at locations distal to the recording electrode and to maintain space clamp. Similarly, we have not addressed changes in potassium currents which are known to occur in the FL model (Bordey and Sontheimer, 1998). We did not see significant changes in membrane resistance with any manipulation; which argues against TC distortion by altered intrinsic membrane properties. Second, it is difficult to ascribe the changes we report solely to the lesion itself rather than the later hyperexcitability that the lesion induces. Increased neuronal activity is known to affect TCs (Takahashi et al., 2010) and regulate GLT-1 function (Yang et al., 2009). Third, there is an interesting discontinuity between the presumably neuroprotective changes reported here (increased neonatal GLT-1 function, increased mature GLT-1-independent function) and previously reported disruptions in glutamate homeostasis reported in the FL model (Campbell and Hablitz, 2005; Dulla et al., 2012). This suggests that there are unexplored elements in ensuring efficacious glutamate homeostasis and in the relationship between glutamate uptake and synaptic glutamate transients. Lastly, our recordings did not discriminate between “reactive” and “non-reactive” astrocytes and therefore our results likely mimic the heterogeneity of astrocytes in a variety of “reactive” states present following FL. Ideally, we would like to identify patch-clamped astrocytes, identify their GFAP expression, and perform anatomical reconstruction of each astrocyte. Unfortunately this has remained technically challenging, and outside the scope of these studies. Investigating these questions and other will require the refinement of methodologies to examine the coupling of astrocytes and neurons on many spatial and temporal scales. Finding solutions to these problems is critical as astrocytes play a major role in the response to brain insult, are altered in diseases of cortical malformation, and offer an unexplored avenue for the development of novel therapeutics.

### Conflict of interest statement

The authors declare that the research was conducted in the absence of any commercial or financial relationships that could be construed as a potential conflict of interest.
